# A One-Phase Injection Method with Dual Inhibition for Improving the Strength and Uniformity of MICP

**DOI:** 10.3390/ma18112514

**Published:** 2025-05-27

**Authors:** Yanni Huang, Fengyin Liu, Xiangtong Zhang

**Affiliations:** 1 School of Civil Engineering and Architecture, Xi’an University of Technology, Xi’an 710048, China; huangyanileehom@163.com (Y.H.); zxt18691767915@163.com (X.Z.); 2 State Key Laboratory of Water Engineering Ecology and Environment in Arid Area, Xi’an University of Technology, Xi’an 710048, China

**Keywords:** microbially induced calcite precipitation, bacterial grouting strategies, low-Ph one-phase injection

## Abstract

The formation and spatial uniformity of calcium carbonate (CaCO_3_) are critical for evaluating the effectiveness of microbial-induced calcium carbonate precipitation (MICP) in geotechnical applications. In recent years, the single-phase injection method has emerged as a promising alternative to traditional two-phase processes by addressing the issue of uneven CaCO_3_ distribution. This study proposes a dual inhibition strategy that delays the mineralization reaction by synergistically lowering pH and temperature, thereby promoting uniform precipitation and enhanced compressive strength in cemented sand columns. A series of experiments, including bacterial growth, aqueous reaction, sand column reinforcement, and microstructural characterization, were conducted. Results show that the minimum pH required for flocculation increases from ~4.5 at 40 °C to ~6.0 at 10 °C. Under dual inhibition, the lag period effectively improved the spatial uniformity of CaCO_3_ and enabled complete calcium utilization within 24 h. After four treatment cycles, the CaCO_3_ content at 10 °C increased by 53%, and the unconfined compressive strength reached 2.5 MPa, a 50% improvement over the 40 °C condition. XRD analysis confirmed that calcite was the dominant phase (85–90%), accompanied by minor vaterite. These findings demonstrate the adaptability and efficiency of the dual inhibition method across temperature ranges, providing a cost-effective solution for broader engineering applications.

## 1. Introduction

Recent advances in MICP technology have been widely reported internationally, covering optimization of microbial strains [1] (Cheng et al., 2019), control of precipitation kinetics [2] (Al Qabany and Soga, 2013), and improvements in treatment uniformity under field conditions [3] (DeJong et al., 2020). Studies such as those by [4] Xiao, Y (2024) have systematically evaluated the relationship between microbial activity, calcium carbonate polymorphs, and mechanical properties, providing critical insights for adaptable MICP applications across diverse terrains. It has shown broad application prospects in many fields such as ground reinforcement, erosion control, and concrete crack repair [4,5,6,7,8,9,10,11,12,13,14,15,16,17,18]. This technology uses urease-producing microorganisms to hydrolyze urea to produce carbonate ions, which react with calcium ions in the solution to form calcium carbonate precipitates, thereby cementing loose sand particles into a whole [19,20,21,22], thereby achieving the purpose of improving soil strength and stability [23,24,25,26]. Although MICP reinforcement has good prospects, the amount of CaCO_3_ generated and its spatial distribution uniformity have a decisive influence on the reinforcement effect.

(1) CO (NH_2_)_2_ + 2H_2_O → 2NH_4_ + CO_3_^2−^ pH↑

(2) Ca + CO_3_^2−^ → CaCO_3_              pH↓

There are still many challenges in the practical application of MICP technology. The traditional multi-phase step-by-step injection process usually requires multiple injections of culture solution and cementing solution alternately, which is not only complicated and cumbersome to operate, but also prone to uneven precipitation. Therefore, in recent years, researchers have explored various strategies to simplify operational procedures and enhance the practical effectiveness of MICP, including optimizing injection techniques [12,27,28,29] and adding specific admixtures [30,31,32]. Despite significant progress, these methods are still far from large-scale industrial field application [33,34,35,36] especially in terms of process simplification and precipitation uniformity control, which need further in-depth research. Microbial-induced calcium carbonate precipitation (MICP) reinforcement usually adopts the traditional two-phase grouting process [37,38,39] to achieve a uniform distribution of calcium carbonate in the soil. However, since the cementing solution injected each time mainly precipitates near the injection port, it often results in more precipitation at the top of the sand column and less precipitation at the bottom, and the strength is distributed in a gradient along the height. Therefore, improving the uniformity of calcium carbonate precipitation after MICP reinforcement has always been an urgent problem to be solved.

In order to solve the above problems, some researchers have proposed a single-phase MICP injection process. This strategy has shown advantages in providing low-cost and high-efficiency MICP [1,39,40,41,42,43]. The key to the single-phase method is to avoid rapid precipitation immediately after the bacterial solution and the binder are pre-mixed to avoid clogging the injection channel. Its principle is to delay the initial precipitation time of CaCO_3_ so that the mixed solution can be transported to a longer distance, thereby achieving uniform precipitation over a larger range. Cheng et al. [1] first proposed a single-phase injection process at a low pH. The pH value of the single-phase slurry (a mixture of bacteria and binder) was lowered before grouting to inhibit the early flocculation of bacteria and the formation of CaCO_3_, and then it was smoothly injected into the soil. This method can not only prevent the clogging of the grouting port but also ensure that the grouting liquid diffuses evenly through the pores of the soil particles, thereby obtaining a consolidated soil with relatively uniform strength. The results of the sand column comparison test show that, compared with the single-phase method without pH adjustment, the CaCO_3_ precipitation distribution in the sand column treated by the low pH single-phase method is more uniform, and the overall strength is significantly improved. After six treatments, the unconfined strength reaches 2.5 MPa, while the conventional method requires more injections to achieve the same strength. In addition, since the single-phase method reduces the volatilization of ammonia, the environmental friendliness is significantly improved. For example, Yang et al. subsequently introduced acetic acid buffer to lower the pH, achieving more and more uniform CaCO_3_ precipitation at the bottom of the sand column, and the strength distribution inside the sand column is more consistent [43]. Subsequently, Lai et al. applied the low pH single-phase method to sand with different particle size gradations and found that the optimal reaction pH of different soil materials is different: the pH in the coarse sand environment is naturally higher, while the pH in the fine sand and tailings sand environment is relatively low [44]. Therefore, it is necessary to adjust the initial pH according to the soil properties to achieve the best uniform reinforcement effect. Overall, the low pH single-phase method significantly improves the utilization rate of calcium ions and improves the uniformity of CaCO_3_ content distribution in various parts. In addition, studies have shown that the use of a single-phase low pH solution in a sterile enzyme-induced precipitation (EICP) system can also improve the conversion rate of calcium ions and make the distribution of CaCO_3_ more uniform along the height of the sand column [34]. In addition to adjusting the pH, the temperature-controlled single-phase injection method provides another idea to improve the uniformity of precipitation: lowering the reaction temperature can delay the CaCO_3_ precipitation process, thereby enhancing the single-phase injection effect. Subsequently, Xiao et al. proposed a temperature-controlled single-phase MICP method: the bacterial solution and the cementing solution were mixed and injected under low temperature conditions, and then the room temperature was restored after the injection was completed to induce CaCO_3_ precipitation [44]. Comparative experiments showed that the CaCO_3_ content in the sand sample treated with this temperature-controlled single-phase method was almost uniform along the height direction, while the sample treated with the traditional room temperature multiphase method formed significantly more precipitation at the top than at the bottom.

Based on the above research, this paper proposes to combine low pH and low temperature to implement “double inhibition” on single-phase slurry. This method can extend the longer lag period of the CaCO_3_ precipitation reaction, thereby further improving the utilization rate of calcium ions and significantly improving the uniformity of CaCO_3_ precipitation distribution after sand column reinforcement. In addition, we determined the optimal reaction temperature through a comprehensive analysis of the results of aqueous solution tests and sand column tests, combined with a microscopic analysis of the generated CaCO_3_ crystals. The experimental results show that compared with previous improvement methods, the “double inhibition” single-phase MICP grouting technology has achieved remarkable results in simplifying the process and improving the reinforcement effect.

## 2. Materials and Methods

### 2.1. Experiment Materials

**Figure 1:**
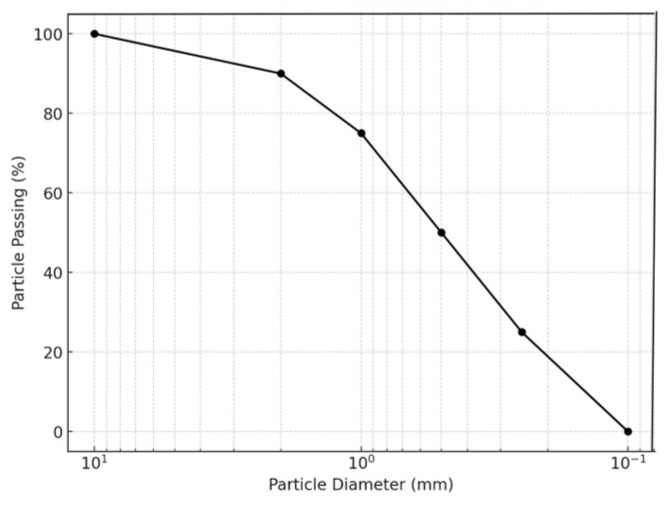
Gradation curve of silica Ottawa sand used in this study.

In this study, ISO standard sand was used as the test aggregate, and its gradation curve is shown in Figure 1. The experimental sand was pure quartz sand (99.7% SiO_2_ mass fraction). After being rinsed with deionized water and dried at 80 °C, the particle size distribution curve shown was obtained according to the standard sieving test. The median particle size and non-uniformity coefficient of the sand indicate that the sand is a poorly graded uniform sand.

The strain used in this study was S. pasteurize DSM 33. The bacteria were cultured in a sterile aerobic medium (200 mL of the culture medium was placed in a 1 L conical flask, shaken at 28 °C and 170 rpm). The culture medium contained 20 g/L yeast extract, 15 g/L ammonium chloride (NH_4_Cl), and 0.1 M nickel chloride (NiCl_2_). The pH was adjusted to 9.25 with 10 M sodium hydroxide (NaOH). The binder solution was a mixed solution of calcium chloride (CaCl_2_) and urea with equimolar concentrations [19,40,45,46,47,48], and its concentration was controlled to be 2 M. A 1.0 M hydrochloric acid (HCl) solution was used to adjust the initial pH value of the mixed solution of the bacterial solution and the binder solution.

### 2.2. Experimental Methods

#### 2.2.1. Bacterial Concentration and Urease Activity

The optical density (OD_600_) of the bacterial solution was measured at a wavelength of 600 nm using a spectrophotometer to determine the bacterial concentration. The initial urease activity was determined using the conductivity method [43]. The steps are as follows: (1) 1 mL of bacterial solution was mixed with 9 mL of 1.11 M urea solution to prepare the test solution; (2) the conductivity change in the solution was monitored at 25 ± 1 °C for 5 min; (3) the urease activity was calculated according to the formula.

The urease activity UA (mmol urea/min) was calculated using the following equation:

(3) $$UA(mM/\min)=\frac{E\times 11.1\times 10}{t}$$

where E is the change rate of electrical conductivity (mS/min); 11.1 is the empirical conversion coefficient correlating conductivity change to urease activity, as established by Hakes et al. [49]; 10 is the dilution factor used when preparing the enzyme solution for testing; and t s the reaction duration (min).

#### 2.2.2. Flocculent Precipitate Testing Method

A series of experiments were conducted to investigate the influence of pH and temperature on Ca^2^⁺-induced bioflocculation by mixing native ureolytic bacterial cultures with CaCl_2_ solutions. The final temperature of the prepared mixtures was set to 10 °C, 25 °C, and 40 °C, respectively, while the CaCl_2_ concentration was maintained at 1 M. The pH of each mixture was adjusted to values ranging from 3.5 to 6.0 using 1 M HCl. The mixtures were left undisturbed for 30 min to allow complete sedimentation of the aggregated bacterial flocs. The residual suspended biomass was quantified by measuring the optical density at 600 nm (OD_600_) of the supernatant, enabling the calculation of the percentage of flocculated bacterial cells.

#### 2.2.3. Calcium Carbonate Content

The acid-washing method was used to evaluate the distribution of CaCO_3_ in the sand column along the depth direction [18]. The specific method is as follows: the treated and solidified sand column is first rinsed with 10 pore volumes (10 PV) of tap water to remove unreacted substances, and then the sand column is taken out and divided into three equal sections along the height direction. After each section of the sand sample is dried at 105 °C, a portion of the sample is taken and acid washed with 1 M hydrochloric acid solution. The weight of the dry sand before and after acid washing is measured, respectively, and the difference is regarded as the mass of precipitated calcium carbonate. The calcium carbonate content (expressed as mass percentage) is defined as the mass of calcium carbonate dissolved by acid washing as a percentage of the dry weight of the sand sample in that section [50,51,52,53,54,55]. Each data point represents the mean value from three replicate tests (n = 3), and standard deviation bars indicate variability. CaCO_3_ content was determined by acid digestion at three vertical segments (top, middle, bottom) of each column and averaged to represent the overall precipitation. UCS was measured on dried whole-column specimens (ϕ = 50 mm, h = 100 mm). It is worth noting that while acid dissolution is effective in quantifying total carbonate precipitates, it may also remove organic biomass or biogenic residues associated with microbial cells. As a result, the reported CaCO_3_ content may be slightly overestimated due to the co-dissolution of non-carbonate materials. This methodological limitation should be considered when interpreting the results. The formula for calculating the amount of CaCO_3_ generated is as follows:

(4) $$\frac{M_{2}-M_{1}}{M_{2}}*100\%$$

where $M_{1}$ is the weight of the dried sample, and$\;M_{2}$ is the weight of the acid-washed sample after drying.

#### 2.2.4. Unconfined Strength

After the sand column was processed, it was demolded and cut into three sections with a height-to-diameter ratio of about 1:1.5 to 1:2 along the height direction. Each sand sample was air-dried for 48 h [47,48,56]. According to the ASTM D2166 standard method, the unconfined compressive strength (UCS) test was carried out on each section of the sand sample at a constant axial loading rate of 1.0 mm/min (ASTM, 2016) [53].

#### 2.2.5. Microscopic Analysis

In order to study the crystal phase and morphology of the CaCO_3_ precipitate formed under the “double inhibition” condition, the product was microscopically analyzed. X-ray diffraction (XRD) analysis was employed to determine the crystal phase composition of CaCO_3_ precipitates, while scanning electron microscopy (SEM) was used to observe the microscopic morphology and cementation characteristics of the mineralized products at different temperatures. The XRD analysis was performed using an XRD-7000 diffractometer (Shimadzu, Japan). SEM observations were conducted using a JSM-6700F field emission scanning electron microscope (JEOL Ltd., Japan), which allows high-resolution imaging with a maximum magnification of up to 650,000×; at the same time, scanning electron microscopy (SEM) was used to observe the microscopic morphology of the precipitation product and examine the cementation interface characteristics between the sand particles.

For both XRD and SEM characterizations, solidified sand column samples were first cut into three segments (top, middle, and bottom), and the middle 2–3 cm portion was used to avoid end effects. For SEM analysis, the selected specimens were oven-dried at 60 °C for 24 h and then gold-coated to enhance conductivity. For XRD, the samples were ground to a fine powder (<75 μm) and mounted using a glass surface to minimize the preferred orientation. All tests were conducted in triplicate to ensure result reproducibility. It should be noted that the XRD analysis conducted in this study was qualitative, primarily for phase identification, rather than quantitative phase analysis. Differences in peak intensities among samples reflect variations in crystal abundance and preferred orientation but do not represent absolute phase quantities.

### 2.3. Test Scheme

#### 2.3.1. Test Tube Test

(1) Ca^2^⁺-induced flocculation characteristics under different pH conditions

In order to study the effect of solution pH on Ca^2^⁺-induced biological flocculation, static experiments of bacterial solution and CaCl_2_ mixed solution were carried out at different temperatures. The bacterial solution was mixed with 2 M CaCl_2_ solution, and a certain amount of hydrochloric acid was used to adjust the initial pH of the mixed solution to 3.5, 4.0, 4.5, 5.0, 5.5, and 6.0, respectively. The initial temperatures were set to 10 °C, 25 °C, and 40 °C, respectively. The prepared mixed solution was allowed to stand for 30 min to allow the generated bacterial flocs to fully settle. Then, the optical density OD_600_ of the supernatant was measured to obtain the concentration of residual bacteria in the suspension, so as to calculate the percentage of flocculated and settled bacteria.

(2) Determination of hysteresis period under low pH and low temperature conditions

To determine the hysteresis time of mineralization reaction under “double inhibition” conditions, 15 mL of bacterial solution pre-cooled to different initial temperatures (10 °C, 25 °C, 40 °C) was quickly mixed with 15 mL of cementing solution (2 M CaCl_2_ and urea solution, pH adjusted to 4) to prepare a single-phase injection solution. The mixed solution was continuously stirred at 400 rpm on a magnetic stirrer, and the change in the solution pH was monitored in real time. When the pH rose to nearly 7, a large amount of flocculent precipitation was observed. At this time, the time experienced by the reaction was recorded, which was the hysteresis period of the mineralization reaction. The measured hysteresis period will be used to select the best injection scheme for subsequent sand column tests.

#### 2.3.2. Sand Column Test

(1) Distribution of bacterial solutions in the sand column under different temperature conditions

**Figure 2:**
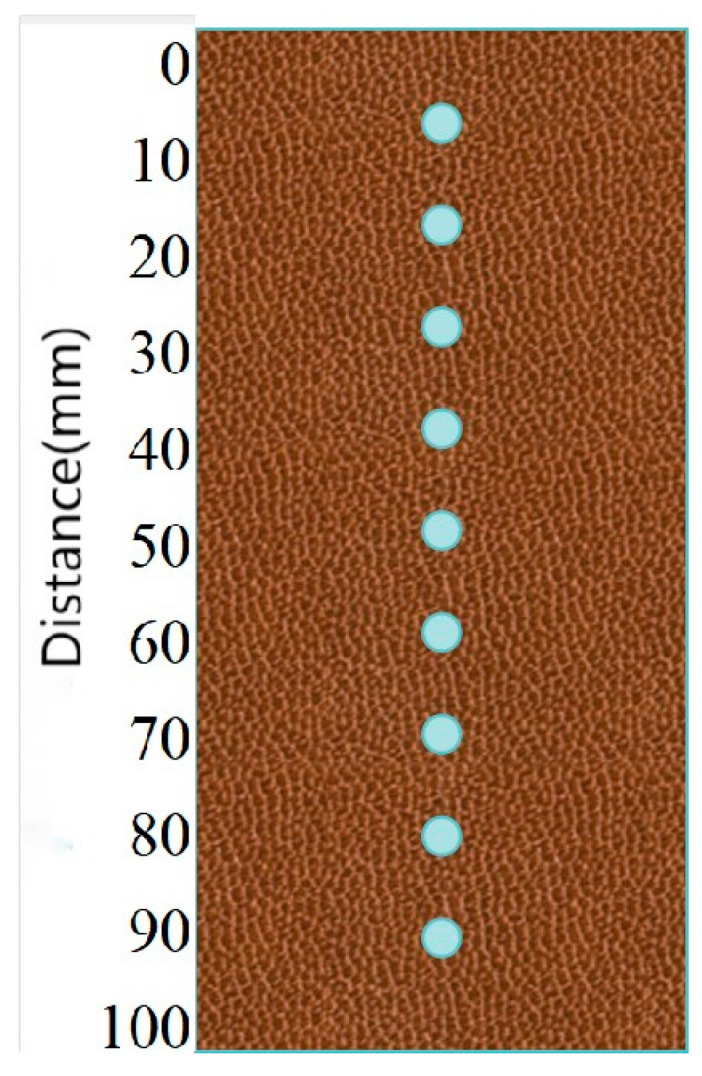
Effect of different temperatures (T) on bacterial distribution. Embedded sampling points in 1m columns.

To study the effect of temperature on the distribution of single-phase injected bacterial solution in the sand column, a comparative test was conducted. The sand column (diameter 39.1 mm, height 100 mm) was prepared using a polyvinyl chloride (PVC) pipe as a mold. To ensure the uniformity of the sand sample, it was divided into three equal parts and then loaded into the mold in turn by dry sand free-fall method to obtain a loose sand column with an initial porosity of about 0.60 and a relative density of about 57%. All tests were repeated three times to ensure the repeatability of the results. Saturated loose sand columns (numbered A and B) were injected with bacterial solution equivalent to 0.6 times pore volume (0.6 PV) and 0.6 PV urea solution (see Table 1 for details), respectively. The concentration of urea solution was 2 M, and its pH was also adjusted to 4 with HCl. Sand column A was injected with a single phase at 25 °C, and sand column B was injected with a single phase at 10 °C. The flow rate was controlled at 5 mL/min during injection, and after filling, the reaction was allowed to proceed without disturbance. After the experiment, the sand column was removed from the device and cut horizontally into three sections along the height direction. Samples were taken from the top, middle, and bottom of each section, for a total of 9 measurement points (see Figure 2). The urea hydrolysis rate of each sample was measured by adding 1 mL of bacterial solution. The urea hydrolysis rate was used as an indicator of the bacterial concentration at that location, and its value was expressed as the ratio of the measured enzyme activity to the initial injected enzyme activity.

(2) Uniformity and strength of sand column reinforcement under different temperature conditions

In order to study the actual reinforcement effect of low temperature and low pH single-phase injection, a sand column reinforcement test with multiple rounds of grouting was carried out. First, the pH of the bacterial solution was pre-adjusted to 4, and then the bacterial solution was mixed with 2 M cementing liquid at a volume ratio of 1:1 under different temperatures (10 °C, 25 °C, 40 °C) and injected into the loose sand column at one time. The reaction was allowed to stand for 24 h as one reinforcement treatment. The above single-phase injection process was repeated until the required number of treatments (N) was reached. After the treatment, the above-mentioned acid washing method and unconfined compressive strength test were used to determine the CaCO_3_ content distribution and compressive strength in the sand column under different temperature conditions. Table 2 lists the specific working parameters of the sand column reinforcement test.

## 3. Results and Discussion

### 3.1. Effect of Dual Inhibition on Calcium Carbonate Precipitation (Aqueous Solution Test)

When 2 M CaCl_2_ was added to the unadjusted pH bacterial solution, a large amount of white flocculent precipitate appeared within seconds, and about 99% of the bacteria precipitated in the form of flocs. This indicates that high concentrations of Ca^2^⁺ can immediately induce bacterial flocculation, which seriously affects the permeability of the solution. With the addition of urea, the pH value will increase [26,28], along with the increase in bicarbonate ion concentration (due to urea hydrolysis), which may lead to irreversible ectopic flocculation and crystal precipitation. Lowering the initial pH of the solution can significantly weaken this rapid flocculation phenomenon: as the pH value decreases, the proportion of white flocculent precipitate produced decreases significantly, while as the temperature increases, the flocculation precipitation increases.

**Figure 3:**
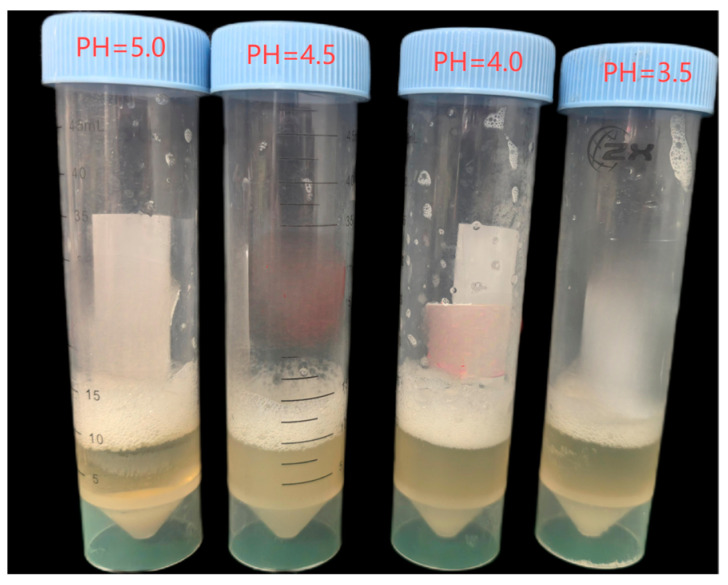
Minimum pH threshold for the formation of flocculent precipitates at 25 °C.

**Figure 4:**
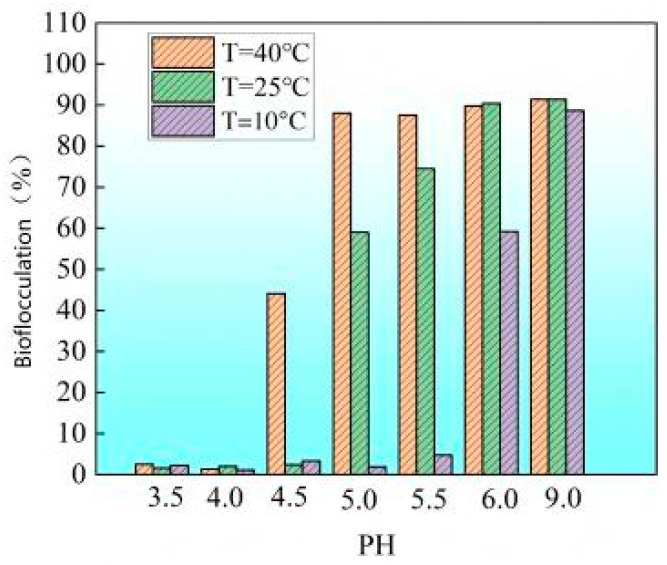
Biomass accumulation of ureolytic bacteria as a function of pH in the presence of 2 M CaCl_2_.

As shown in Figure 3, using ambient temperature (T = 25 °C) as a representative example, it was observed that when the pH value was reduced below approximately 5.0, the initially formed white flocculent precipitates almost completely dissolved, resulting in negligible CaCO_3_ precipitation. In contrast, when the pH reached 5.0, a significant amount of flocculent precipitate began to appear, indicating that the threshold pH for extensive precipitation at 25 °C is approximately 5.0. This phenomenon suggests that maintaining a lower pH (<5.0) under ambient conditions facilitates the extended transport of bacterial suspensions within sand columns, effectively preventing clogging near the injection inlet due to premature flocculation [54]. In contrast, as shown in Figure 4, at a higher temperature T = 40 °C, even if the pH is lowered to 4, a small amount of flocculent precipitation will still be produced, indicating that the low pH needs to be more strictly controlled at high temperatures to inhibit early flocculation. From the above results, it can be seen that in order to ensure that the solution injection is completed before flocculation occurs, a sufficiently long lag period must be provided.

**Figure 5:**
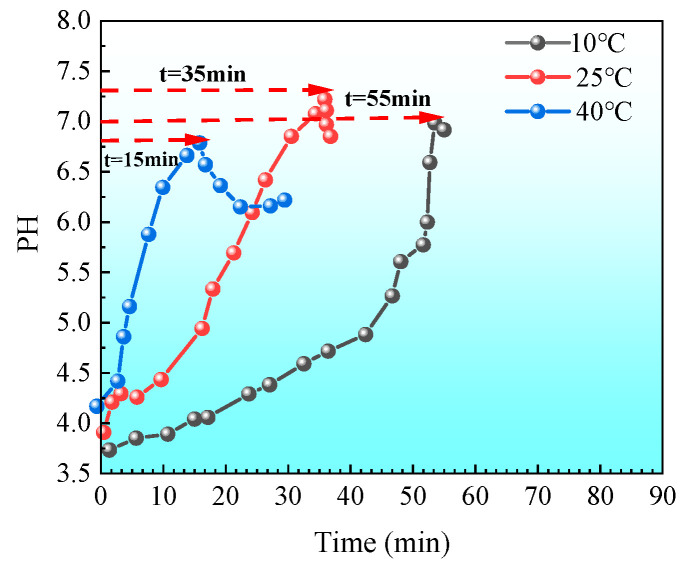
Lag time under different temperatures at an initial pH of 4.

**Figure 6:**
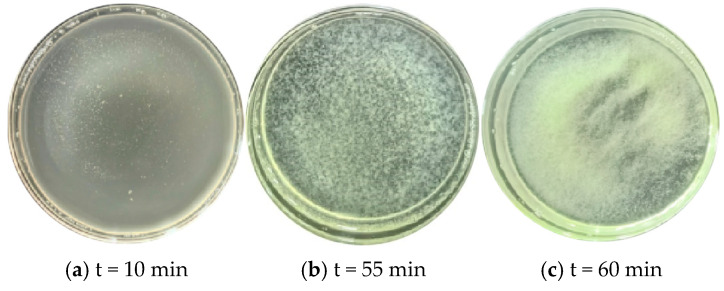
Real-time change in solution pH at an initial pH of 4 and temperature of 10 °C.

As the reaction proceeds, the pH of the solution will gradually rise. As shown in Figure 5, under the condition of initial pH = 4, there are significant differences in the rate of increase in the solution pH at different temperatures: the higher the initial temperature, the faster the pH rises; the lower the initial temperature, the slower the pH rises. This is mainly attributed to the strong activity of bacterial urease at high temperatures, which accelerates the hydrolysis of urea, thereby causing the pH to rise rapidly. When the initial temperature is 40 °C, the solution pH rises from 4 to about 7 in only about 10 min; while when the initial temperature is 10 °C, it takes about 60 min to reach the same pH. It can be seen that lowering the culture temperature can significantly prolong the induction period of the mineralization reaction, This temperature dependence is consistent with the findings of Hosseini S M J et al. (2019) [54], who reported that lower temperatures inhibit metabolic activity, thereby delaying the onset of microbial-induced nucleation. For example, as shown in Figure 6, when the initial conditions are pH = 4 and T = 10 °C, the flocculation phenomenon is inhibited for about 60 min; and when the initial temperature is increased to 40 °C, the time for flocculation to be inhibited is greatly shortened (less than 10 min). This trend is consistent with the results of the aforementioned aqueous solution test, indicating that a higher pH threshold is required to induce flocculation at lower temperatures.

**Figure 7:**
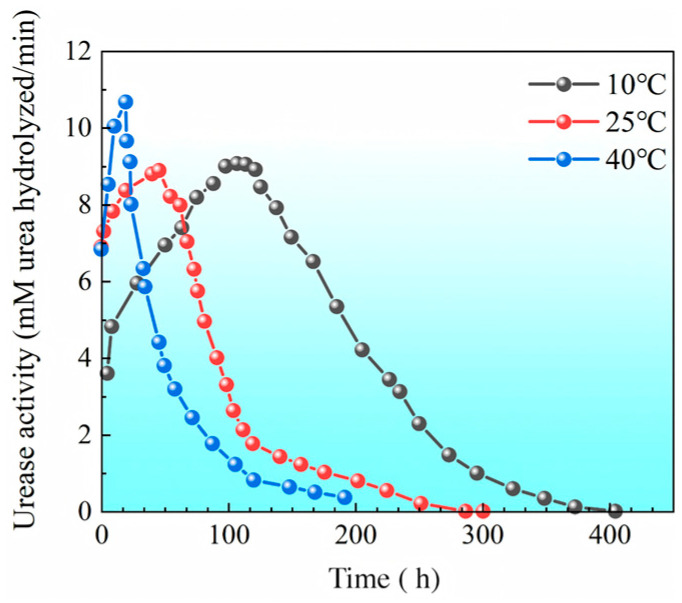
Urease activity of bacteria over time under different temperature conditions.

Figure 7 shows the relationship between the urease activity of bacteria and time under different temperature conditions. During the initial 20 h, urease activity increased at both 40 °C and 25 °C. Around 50 h after the start of the experiment, urease activity at 40 °C and 25 °C reached a peak and then declined rapidly. In contrast, at 10 °C, urease activity continued to increase and peaked between 50 and 120 h, followed by a general decline. The higher the temperature, the faster the decrease in urease activity. After approximately 200 h, urease activity at 40 °C dropped to nearly zero, indicating that most bacteria either died or lost urease activity at this temperature. However, at 10 °C, urease activity did not decrease to near zero until approximately 400 h. Clearly, the lower the experimental temperature, the longer urease activity is sustained, resulting in a prolonged mineralization reaction period.

The above results are of great significance for practical applications: in low-temperature winter environments, if reinforcement requires a shorter lag period, there is no need to adjust the pH of the bacterial solution too low, which can save economic and time costs while ensuring uniform injection. Similarly, in high-temperature summer environments, the initial pH should be appropriately lowered according to the required lag period to meet engineering specifications. By reasonably controlling the temperature and pH to achieve a balance between the lag period and environmental conditions, it can ensure that the MICP slurry is fully infiltrated and dispersed before entering the reaction stage, thereby improving the uniformity of precipitation and reinforcement effect.

### 3.2. Effect of Low Temperature and Low pH Single-Phase Method on MICP Cementation (Sand Column Test)

#### 3.2.1. Distribution of Urease Activity in Sand Columns

**Figure 8:**
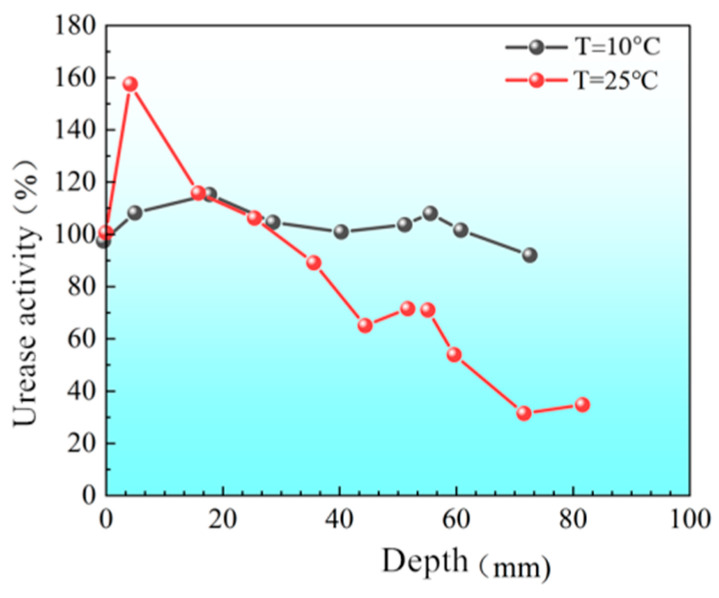
Spatial distribution of urease activity within sand columns at low pH and different temperatures.

Figure 8 shows the distribution of urease activity along the depth of sand columns treated at different initial temperatures under low initial pH conditions (initial pH = 4). The results indicate significant variability in urease activity at different depths under room temperature (T = 25 °C), with a standard deviation of approximately 41.3. In contrast, at a lower temperature (T = 10 °C), urease activity across various depths was more consistent, with a standard deviation of only about 14.0. A more uniform distribution of urease activity within the sand columns implies a more uniform bacterial deposition throughout the column. Thus, the sand column treated at lower temperatures (Column B) demonstrated significantly more uniform urease activity, attributed to bacteria maintaining enzyme activity for a longer duration during nutrient-depleted dormancy periods at lower temperatures. This conclusion aligns with the results from bacterial storage experiments, indicating that the lower the experimental temperature, the longer the duration of bacterial enzyme activity. Under identical pH conditions, lowering the initial temperature of the bacterial suspension temporarily reduces urease activity and slows the rate of urea hydrolysis [27], thereby prolonging bacterial viability and enabling the bacterial solution to travel farther within the sand column. Completing the injection process at T = 10 °C results in a more uniform bacterial (enzyme activity) distribution within the sand columns, thereby facilitating subsequent uniform precipitation.

#### 3.2.2. Calcium Carbonate Content and Unconfined Strength of Sand Columns

**Figure 9:**
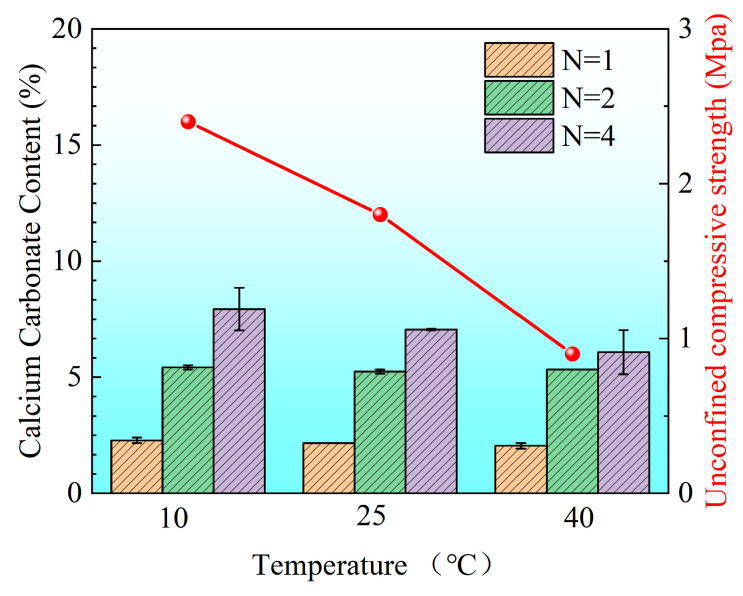
Calcium carbonate content and unconfined compressive strength (UCS) of sand columns after four MICP treatment cycles (N = 4) under curing temperatures of 10 °C, 25 °C, and 40 °C.

Figure 9 compares the average CaCO_3_ content and unconfined compressive strength (UCS) of sand columns subjected to different numbers of treatment cycles (N) at various temperatures. It is worth noting that when one-phase injection was conducted at a room temperature without pH adjustment, severe clogging occurred at the top of the sand column, preventing effective cementation at the bottom. Consequently, this group failed to form complete specimens, and their strength values are not reported in the figure.

Experimental results demonstrate that UCS increases significantly with the number of treatment cycles. As N increased from 2 to 4, the total CaCO_3_ content exhibited an upward trend with a decreasing temperature. This finding aligns with prior studies indicating that lower temperatures can prolong urease activity and enhance calcium ion utilization efficiency, leading to more effective mineralization [54,55]. Moreover, the spatial uniformity of CaCO_3_ precipitation became more pronounced under low-temperature conditions, particularly after multiple reinforcement cycles.

After four treatments, the UCS of the sand column cured at 10 °C reached approximately 2.4 MPa, surpassing that of the specimens treated at 25 °C and 40 °C. Notably, although the UCS of samples treated at 40 °C was slightly higher than that of the 25 °C group after fewer treatment cycles, this trend reversed after the fourth treatment. This observation suggests that, while the total CaCO_3_ content initially dominates strength development, the uniformity of precipitation plays an increasingly critical role during repeated treatments, consistent with the findings of Zhu et al. (2025) [57].

**Figure 10:**
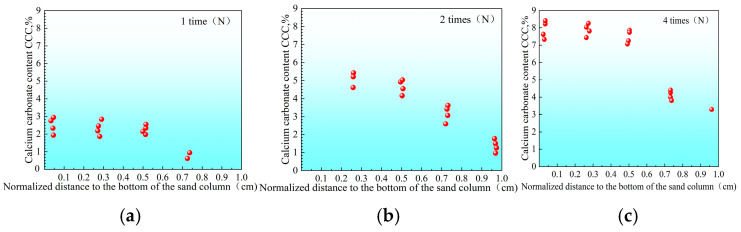
Distribution of calcium carbonate in sand column without adjusting pH and treatment times ((**a**) 1, (**b**) 2, and (**c**) 4 cycles).

**Figure 11:**
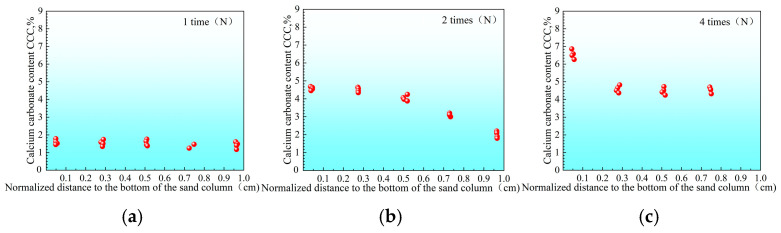
Distribution of calcium carbonate within sand columns at 40 °C after different numbers of treatments ((**a**) 1, (**b**) 2, and (**c**) 4 cycles).

**Figure 12:**
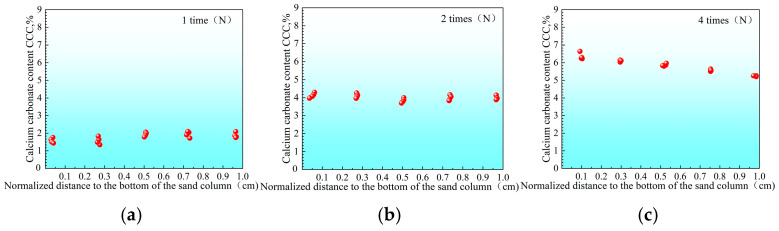
Distribution of calcium carbonate within sand columns at 25 °C after different numbers of treatments ((**a**) 1, (**b**) 2, and (**c**) 4 cycles).

**Figure 13:**
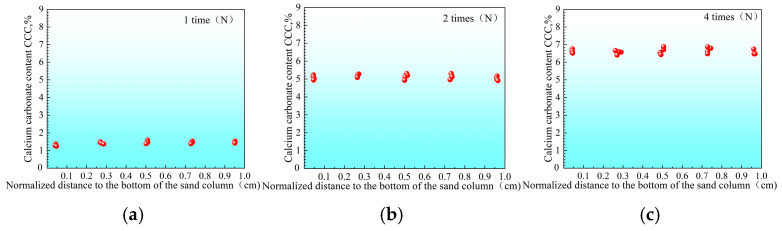
Distribution of calcium carbonate within sand columns at 10 °C after different numbers of treatments ((**a**) 1, (**b**) 2, and (**c**) 4 cycles).

The distribution of CaCO_3_ content at different depths of the sand column was further analyzed (Figure 10, Figure 11, Figure 12 and Figure 13). It can be seen that there are significant differences in the distribution of CaCO_3_ along the height in the sand column under different reinforcement conditions. In the two-phase reinforced sample without adjusting the pH (Figure 10), CaCO_3_ is mainly concentrated in the upper part of the sand column; with the increase in the number of reinforcement times, the degree of uneven distribution is further aggravated, and the CaCO_3_ content at the bottom is always at a low level. This phenomenon explains why the traditional two-phase sample is prone to insufficient strength in the lower part: due to insufficient calcium carbonate precipitation at the bottom, the structure may be unbalanced or have concentrated stress. Similar trends have also been reported by Liu et al. (2025) [58], who noted that this phenomenon explains why the traditional two-phase sample is prone to insufficient strength in the lower part: due to insufficient calcium carbonate precipitation at the bottom, the structure may be unbalanced or stress concentrated. Early-stage precipitation tends to occur near the injection point under static flow conditions, leading to poor cementation in deeper layers.

In contrast, under the low-pH single-phase treatment, the lower the temperature, the more uniform the precipitation distribution. For instance, after four reinforcements at a high temperature of 40 °C (Figure 11), the CaCO_3_ distribution in the sample is still somewhat uneven, and the content in the middle and bottom is slightly lower. However, under room temperature of 25 °C (Figure 12) and low temperature of 10 °C (Figure 13), the CaCO_3_ distribution of the reinforced specimens was relatively uniform. Xiao et al. (2021) similarly observed that lower temperatures not only delayed the onset of ureolytic activity but also facilitated a more homogeneous spatial distribution of biominerals [44].

The coefficient of variation in the CaCO_3_ content was the lowest under the low temperature condition of 10 °C, and the precipitation was almost uniform along the height. This difference may be related to the effect of temperature on the morphology and nucleation growth of CaCO_3_ crystals (see Section 3.3 for details), which has also been discussed in recent studies focusing on temperature-controlled MICP processes for geotechnical applications.

It is worth noting that from the second to the fourth grouting treatment, although the total CaCO_3_ content of each group of samples increased, the increase was smaller than the increase from the first to the second grouting (this trend is also reflected in the coefficient of variation analysis). The reason for this phenomenon may be that the precipitation products injected early occupied part of the pore space, making it impossible for the subsequent grouting solution to fully penetrate to the bottom of the sand column, resulting in the new precipitation in the subsequent reinforcement process mainly concentrated in the upper part, exacerbating the uneven deposition inside the sample.

**Figure 14:**
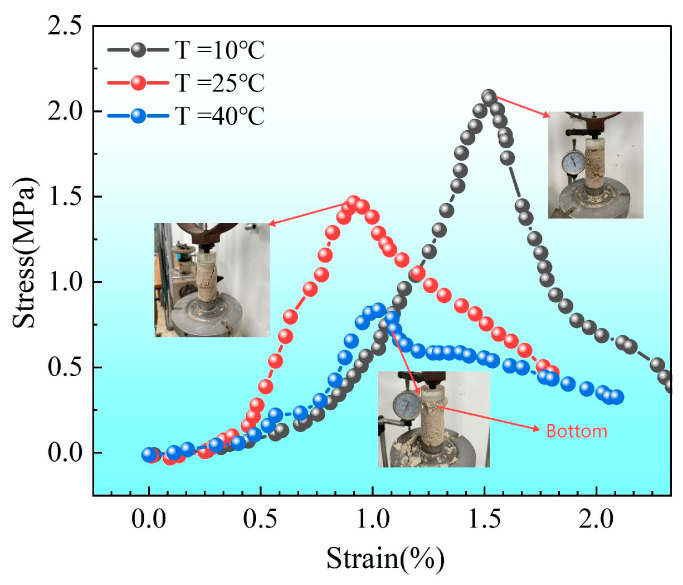
Typical stress–strain curves and failure modes of sand columns treated four times (N = 4) at different temperatures (10 °C, 25 °C, and 40 °C). Arrows indicate the correspondence between each stress–strain curve and its respective fractured specimen image. (10 °C is tensile failure, 25 °C is shear failure, and 40 °C is bottom failure).

Figure 14 shows typical stress–strain curves and failure modes of sand columns treated four times (N = 4) at different temperatures. During unconfined compression testing, samples at all temperatures underwent an initial compaction stage followed by a linear elastic deformation stage, with minimal differences in stiffness observed during this elastic stage. When stress reached peak strength, the elastic stage ended, and cracks began to appear. The samples treated at the lower temperature (T = 10 °C) exhibited a longer elastic stage and higher peak strength, likely due to the higher CaCO_3_ content and more uniform distribution under these conditions. After reaching peak strength, cracks rapidly propagated until complete failure, and all sand column specimens exhibited brittle failure characteristics. However, the failure locations varied depending on the conditions. At high temperatures (40 °C), failure mainly occurred at the bottom of the specimens due to the relatively lower CaCO_3_ content. In contrast, samples treated at room temperature (25 °C) and low temperature (10 °C) exhibited vertical splitting failures (vertical cracks along the column height), indicating a relatively uniform distribution of precipitation and better structural integrity under these two conditions. This finding is consistent with the analysis presented previously in Figure 12.

Figure 14 shows the typical stress–strain curves and failure modes of sand columns treated four times (N = 4) at different temperatures (10 °C, 25 °C, and 40 °C). Arrows indicate the correspondence between each stress–strain curve and its respective fractured specimen image. The top surface of the sample is marked in each image. Specimens treated at lower temperatures exhibited higher peak strength and better ductility. At 40 °C, failure initiated at the bottom part of the column, likely due to insufficient calcium carbonate precipitation, leading to reduced mechanical integrity. In contrast, columns cured at 10 °C and 25 °C showed vertical splitting failures, consistent with a more uniform cementation structure.

It should be noted that the stress–strain curves in Figure 14 represent individual specimens, whereas the compressive strength values reported in Figure 9 are averaged over three replicates. The relatively low strength of the 40 °C sample in Figure 14 (<1 MPa) is attributed to nonuniform calcium carbonate distribution, as evidenced by early failure from the sample base. This is also visually supported by the failure image.

In order to quantify the effect of the inhomogeneity of crystal spatial distribution on the structural strength, an index parameter is defined to characterize the crystal distribution characteristics. The smaller the value of this parameter, the more uniform the crystal distribution in the structure; on the contrary, the larger the value, the more uneven the crystal distribution. The calculation formula for this index parameter is as follows:

(5) $$C_{f}=\frac{\Delta C}{\Delta C_{d}}=\frac{C_{\max}-C_{\min}}{\Delta C_{d}}$$

$C_{\max}$ represents the maximum calcium carbonate content along the vertical direction of the sand column under the same number of treatment cycles; $C_{\min}$ represents the minimum calcium carbonate content measured along the vertical direction under the same number of treatment cycles; $\Delta C_{d}$ represents the maximum difference in calcium carbonate content along the vertical direction under the same number of treatment cycles.

Based on the data presented in Figure 11, Figure 12 and Figure 13, the sample with a compressive strength of 1.3 MPa exhibited the largest difference in CaCO_3_content, with $\Delta C_{d}=2.3\%$. Using Equation (5), the calculated values of the inhomogeneity index $C_{f}$ for each treatment condition are summarized in Table 3.

From Table 3, we can see that:

1. From the perspective of the uneven characteristic index ($C_{f}$) of the spatial distribution of calcium carbonate, the values ($C_{f}$) of each group (respectively, 1.0, 0.25, and 0.065) are all greater than zero, which shows that although the crystal content has gradually increased, the spatial distribution of calcium carbonate has not reached a completely uniform state as a whole.
2. For different spatial distributions of calcium carbonate (different $C_{f}$), the structural strength shows obvious differences. Under the premise that the average % of calcium carbonate content is similar, as the uniformity of calcium carbonate distribution increases ($C_{f}$ becomes smaller), the structural strength increases significantly. For example, when it decreases from 1.0 to 0.25 and further to 0.065, the average content of calcium carbonate increases from 5.93% to 6.50%, and the strength of the corresponding structure increases significantly (from 1.3 MPa to 2.5 MPa). This shows that the more uniform (the $C_{f}$ smaller) the spatial distribution of calcium carbonate is, the more uniform the cementation effect of the structure is, which is conducive to significantly improving the overall strength of the structure.
3. From the perspective of calcium carbonate content, under certain conditions (the degree of spatial distribution uniformity is close), increasing the calcium carbonate content is obviously conducive to improving the structural strength. For example, when the $C_{f}$ decreases from 0.25 to 0.065, the average content of calcium carbonate only increases from 6.25% to 6.50%, but the structural strength increases from 1.7 MPa to 2.5 MPa, which shows that even if the difference $C_{f}$ is not large, a small increase in the content of calcium carbonate can still significantly enhance the bearing capacity of the structure. This is consistent with the principle that after the calcium carbonate content increases, the internal bonding and filling effect of the structure are enhanced, the integrity of the structure is improved, and thus the structural strength is improved.

In summary, this experimental result clearly reflects the joint influence of crystal spatial distribution and crystal content on structural strength. Under the premise of ensuring a certain crystal content, improving the uniformity of crystal spatial distribution (reducing) is significantly beneficial to improving structural strength; under the premise of relatively uniform crystal spatial distribution, increasing the crystal content can also effectively improve structural strength. This is consistent with the conclusions in the previous analysis that the uniform distribution of crystals improves structural heterogeneity and the crystal content improves the bonding and filling effect of the structure. This further confirms the regularity of the influence of crystal content and crystal spatial distribution on structural strength.

### 3.3. Microstructural Characteristics and XRD Analysis

**Figure 15:**
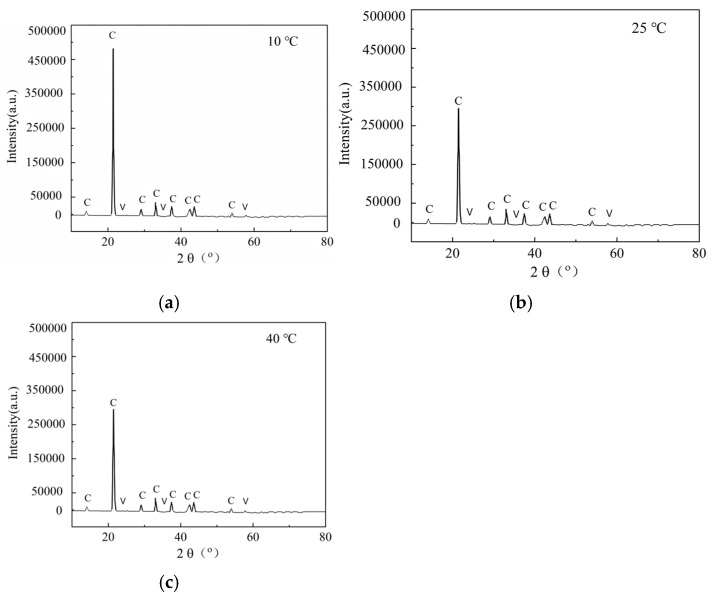
XRD analysis of biocemented samples: (**a**) 10 °C, (**b**) 25 °C, (**c**) 40 °C. C represents calcite, V represents vaterite.

Figure 15 presents the qualitative XRD patterns of CaCO_3_ precipitates collected from sand columns subjected to four treatment cycles (N = 4) at different temperatures (10 °C, 25 °C, and 40 °C). Samples were extracted from the middle section of each column in accordance with the standardized protocol described in Section 2.2.5, involving vertical segmentation of the column and oven-drying prior to characterization. The major diffraction peaks observed across all temperature conditions are consistent in position, corresponding to the characteristic planes of calcite, thereby confirming that calcite is the dominant crystalline phase under the experimental conditions. Differences in peak intensities were evident, suggesting variations in crystallinity or relative abundance of the CaCO_3_ precipitates among temperature groups. Specifically, samples cured at lower temperatures exhibit sharper and more intense calcite peaks, suggesting a higher degree of crystallinity and possibly larger crystal sizes. Conversely, samples at higher temperatures present relatively weaker calcite peaks with the appearance of minor vaterite signals [59], indicating less stable crystallization pathways and a higher proportion of metastable phases. This difference in crystallinity and phase composition is critical, as it directly influences the mechanical properties of the biocemented sand columns. All XRD patterns were plotted using uniform *y*-axis scaling to facilitate direct visual comparison. It is important to note that this is a qualitative XRD analysis. Although minor peaks possibly attributable to vaterite were occasionally detected at localized sampling depths, they were weak and not consistently present across all specimens.

**Figure 16:**
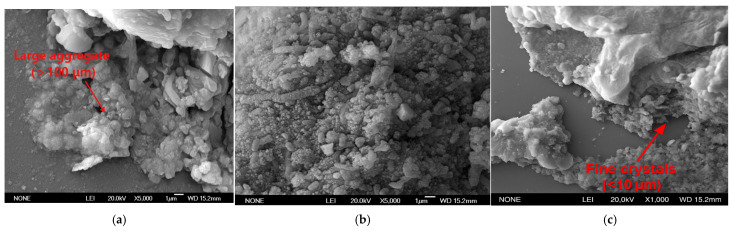
SEM of sand sample treated at ((**a**–**c**) 10 °C, 25 °C, 40 °C).

Figure 16 shows the SEM micromorphology of CaCO_3_ precipitates obtained at 10 °C, 25 °C and 40 °C. It can be observed that the morphology and size of CaCO_3_ crystals precipitated under different temperature conditions are different: the powdery precipitate consists of irregular particles overall, while the morphology of CaCO_3_ crystals deposited on the surface of sand grains is relatively consistent. As the temperature increases, crystal size decreases significantly. At lower temperatures (10 °C and 25 °C), some larger blocky CaCO_3_ crystals appeared in the sample (both on the surface of sand grains and in free powder) [60,61]; while the CaCO_3_ particles formed at high temperatures of 40 °C are generally less than 10 μm in size. In contrast, the size of the crystals generated at 10 °C increases significantly, and the diameter of some particles exceeds 100 μm, which is about 10 times the size of the crystals at 40 °C. This shows that the increase in temperature inhibits the growth process of CaCO_3_ crystals, causing the precipitation to exist in the form of smaller grains; while lower temperatures are conducive to the formation of larger CaCO_3_ crystals. Although the crystal size and morphology change with temperature, as shown in the above XRD results, the mineral phase precipitated at each temperature is calcite [62,63], which explains why the strength can still perform well despite the large crystals under low-temperature conditions: the large and evenly distributed calcite crystals form effective cementation between the particles, improving the overall strength and stability of the sand column.

### 3.4. Practical Considerations for Field Implementation

Although the present study provides a promising strategy for improving the uniformity and strength of MICP-treated sand columns via a dual-inhibition approach, it is important to recognize the limitations of using clean, homogeneous quartz sand under controlled laboratory conditions. In practical field applications, the method would need to be adapted to more complex terrains, such as silty sands, mixed-grain soils, or weakly cohesive deposits. Therefore, future research should focus on validating this technique under heterogeneous subsurface conditions, accounting for variations in pore structure, permeability, and mineralogical composition. Additionally, the implementation of this method in the field could involve surface grouting, deep well injection, or infiltration trenches, with real-time monitoring of pH and temperature to fine-tune the lag period. Seasonal and climatic factors should also be considered, as they influence the optimal pH thresholds and urease activity. The flexibility of this approach in adjusting to different field conditions makes it a promising candidate for foundation reinforcement, slope stabilization, and erosion control in variable geotechnical settings.

## 4. Conclusions

Microbial-induced calcium carbonate precipitation (MICP) technology has great application potential in foundation reinforcement, but there is still room for improvement in strengthening the cementation process. This paper proposes and verifies a low-temperature and low-pH “double inhibition” MICP single-phase grouting method to improve the uniformity and strength of MICP reinforcement. Through bacterial culture, biochemical precipitation tests, sand column reinforcement tests, and XRD microanalysis, the influence of low temperature and low pH conditions on the MICP process was systematically studied, and the following main conclusions were obtained:

1. “Double inhibition” significantly improves precipitation uniformity: The double inhibition of low temperature (10 °C) and low pH (pH = 4) extends the lag period of the MICP reaction to about 60 min, promotes the uniform penetration of bacteria and cementing fluid in the sand column, and reduces accumulation deposition at the injection port. After double inhibition treatment, the coefficient of variation in the Ca CO_3_ content in the sand column along the height decreased from 0.12 at 40 °C to 0.03 at 10 °C, and the precipitation uniformity increased by about four times.
2. Dynamic transformation of the relationship between strength and number of treatments: The compressive strength of the sand column increased significantly with the increase in the number of treatments (the peak strength was about 2.4 M Pa after four treatments). The initial strength was high at a high temperature of 40 °C, but after four treatments, the strength of the sand column was higher at low temperature (10 °C) and room temperature (25 °C). This shows that with the increase in the number of treatments, the dominant factor affecting the strength of the sand column changes from the amount of Ca CO_3_ precipitation to the uniformity of precipitation distribution: the early reinforcement strength is mainly affected by the amount of generation, while the spatial consistency of precipitation after multiple reinforcements contributes more significantly to the strength.
3. Crystal morphology and environmental conditions: XRD analysis shows that the cement generated under various temperature conditions is mainly calcite as the main crystal form, accompanied by a small amount of vaterite. The crystal morphology of CaCO_3_ crystals is less affected by temperature, and the crystal phase composition remains consistent at different temperatures. However, the temperature has a significant effect on crystal size: some large-sized calcite crystals are formed under low-temperature conditions, while the precipitation under high-temperature conditions is mainly fine grains, but these differences do not change the cementing effect of precipitation on sand particles.
4. Economy and seasonal adaptability: In low-temperature winter environments, by adjusting the pH and temperature of the bacterial solution, the required minimum pH threshold can be increased to about 6 to obtain a sufficient lag period and reduce the risk of uneven reinforcement; in high-temperature summer environments (such as T = 40 °C), the pH threshold should be reduced to about 4.5 to ensure the required lag period. This strategy of flexibly adjusting parameters according to seasonal temperature changes can save time and reagent costs while ensuring reinforcement effects, providing higher economy and adaptability for field applications of MICP technology.

## Figures and Tables

**Table 1:** Composition and conditions of injection solutions in sand columns A and B (single-phase injection volume is 0.6 PV).

Sand Column A		Sand Column B	
0.6 PV bacterial solution (PH = 4)	0.6 PV urea (2 M)	0.6 PV bacterial solution (PH = 4)	0.6 PV urea (2 M)
T = 25 °C	T = 25 °C	T = 10 °C	T = 10 °C

**Table 2:** Sand column test condition.

Serial Number	CS (M)	PH	Temperature (°C)	Treatment Cycles (N)		
1#	2.0	4	10	1	2	4
2#	2.0	4	25	1	2	4
3#	2.0	4	40	1	2	4

**Table 3:** Results of the $C_{f}$ Index parameter under different strengths.

UCS (MPa)	1.3 MPa	1.7 MPa	2.5 Mpa
Inhomogeneous characteristic index $\left(C_{f}\right)$	1.0	0.25	0.065
Calcium carbonate content CCC%	5.93	6.25	6.5

## Data Availability

The original contributions presented in this study are included in the article. Further inquiries can be directed to the corresponding author.

## References

[1] Cheng L., Shahin M.A., Chu J. (2019). Soil bio-cementation using a new one-phase low-pH injection method. Acta Geotech..

[2] Al Qabany A., Soga K. (2013). Effect of chemical treatment used in MICP on engineering properties of cemented soils. Géotechnique.

[3] DeJong J.T., Soga K., Kavazanjian E., Burns S., Van Paassen L.A., Al Qabany A., Aydilek A., Bang S.S., Burbank M., Caslake L.F. (2020). Biogeochemical processes and geotechnical applications: Progress, opportunities and challenges. Géotechnique.

[4] Xiao Y., Cui H., Zaman M., Shi J., Wu H. (2024). Constitutive modeling for biocemented calcareous sands. Int. J. Geomech..

[5] Wang Y., Konstantinou C., Tang S., Chen H. (2023). Applications of microbial-induced carbonate precipitation: A state-of-the-art review. Biogeotechnics.

[6] Hammes F., Seka A., de Knijf S., Verstraete W. (2003). A novel approach to calcium removal from calcium-rich industrial wastewater. Water Res..

[7] Chen F., Deng C., Song W., Zhang D., Al-Misned F.A., Mortuza M.G., Gadd G.M., Pan X. (2016). Biostabilization of desert sands using bacterially induced calcite precipitation. Geomicrobiol. J..

[8] Bang S.S., Galinat J.K., Ramakrishnan V. (2001). Calcite precipitation induced by polyurethane-immobilized *Bacillus pasteurii*. Enzym. Microb. Technol..

[9] Choi S.G., Chang I., Lee M., Lee J.H., Han J.T., Kwon T.H. (2020). Review on geotechnical engineering properties of sands treated by microbially induced calcium carbonate precipitation (MICP) and biopolymers. Constr. Build. Mater..

[10] Feng D., Wang Y., Chen D., Liang S. (2024). Experimental study on the influence mechanism of clay particles on the microbial treatment of granite residual soil. Constr. Build. Mater..

[11] Fu T., Saracho A.C., Haigh S.K. (2023). Microbially induced carbonate precipitation (MICP) for soil strengthening: A comprehensive review. Biogeotechnics.

[12] Liu S., Wang R., Yu J., Peng X., Cai Y., Tu B. (2020). Effectiveness of the anti-erosion of an MICP coating on the surfaces of ancient clay roof tiles. Constr. Build. Mater..

[13] Xiao Y., He X., Zaman M., Ma G., Zhao C. (2022). Review of strength improvements of biocemented soils. Int. J. Geomech..

[14] Xiao Y., Zhang Z., Stuedlein A.W., Evans T.M. (2021). Liquefaction modeling for biocemented calcareous sand. J. Geotech. Geoenviron. Eng..

[15] Yu K., Ran Y., Shi J., Duan M., Ouyang Z. (2025). Physical property of MICP-treated calcareous sand under seawater conditions by CPTU. Biogeotechnics.

[16] Zhang J., Xiao Y., Liu H., Chu J. (2024). Role of bacteria on bio-induced calcium carbonate formation: Insights from droplet microfluidic experiments. Géotechnique.

[17] Zhang X., Wang H., Wang Y., Wang J., Cao J., Zhang G. (2024). Improved methods, properties, applications and prospects of microbial induced carbonate precipitation (MICP) treated soil: A review. Biogeotechnics.

[18] Zhang Y., Liu Y., Sun X., Zeng W., Xing H., Lin J., Kang S., Yu L. (2024). Application of microbially induced calcium carbonate precipitation (MICP) technique in concrete crack repair: A review. Constr. Build. Mater..

[19] Cheng L., Kobayashi T., Shahin M.A. (2020). Microbially induced calcite precipitation for production of “bio-bricks” treated at partial saturation condition. Constr. Build. Mater..

[20] DeJong J.T., Fritzges M.B., Nüsslein K. (2006). Microbially induced cementation to control sand response to undrained shear. J. Geotech. Geoenviron. Eng..

[21] Ivanov V., Chu J. (2008). Applications of microorganisms to geotechnical engineering for bioclogging and biocementation of soil in situ. Rev. Environ. Sci. Biotechnol..

[22] Nemati M., Voordouw G. (2003). Modification of porous media permeability using calcium carbonate produced enzymatically in situ. Enzym. Microb. Technol..

[23] Wu C., Chu J., Cheng L., Wu S. (2019). Biogrouting of aggregates using premixed injection method with or without pH adjustment. J. Mater. Civ. Eng..

[24] Ma G., He X., Xiao Y., Chu J., Liu H., Stuedlein A.W., Evans T.M. (2023). Spatiotemporal evolution of biomineralization in heterogeneous pore structure. Can. Geotech. J..

[25] Zhang J., Yin Y., Shi W., Bian H., Shi L., Wu L., Han Z., Zheng J., He X. (2023). Strength and uniformity of EICP-treated sand under multi-factor coupling effects. Biogeotechnics.

[26] Zheng J., Lai H., Cui M., Ding X., Weng Y., Zhang J. (2023). Biogrouting technologies for enhancing uniformity of biocementation: A review. Biogeotechnics.

[27] Martinez B.C., DeJong J.T., Ginn T.R., Montoya B.M., Barkouki T.H., Hunt C., Tanyu B., Major D. (2013). Experimental optimization of microbial-induced carbonate precipitation for soil improvement. J. Geotech. Geoenviron. Eng..

[28] Omoregie A.I., Ong D.E.L., Li P.Y., Senian N., Hei N.L., Esnault-Filet A., Muda K., Nissom P.M. (2024). Effects of push-pull injection–suction spacing on sand biocementation treatment. Geotech. Res..

[29] Zhao C., Xiao Y., He X., Liu H., Liu Y., Chu J. (2025). Influence of injection methods on bio-mediated precipitation of carbonates in fracture-mimicking microfluidic chip. Géotechnique.

[30] Ma G., He X., Jiang X., Liu H., Chu J., Xiao Y. (2021). Strength and permeability of bentonite-assisted biocemented coarse sand. Can. Geotech. J..

[31] Xiao Y., He X., Evans T.M., Stuedlein A.W., Liu H. (2019). Unconfined compressive and splitting tensile strength of basalt fiber-reinforced biocemented sand. J. Geotech. Geoenviron. Eng..

[32] Xu X., Guo H., Li M., Fu H. (2022). Improving microbially induced calcium carbonate precipitation effects by nacre extractions. Geotech. Lett..

[33] Cui M.-J., Chu J., Lai H.-J. (2024). Optimization of one-phase-low-pH enzyme-induced carbonate precipitation method for soil improvement. Acta Geotech..

[34] Li Y., Guo Z., Wang L., Yang H. (2023). A coupled bio-chemo-hydro-wave model and multi-stages for MICP in the seabed. Ocean Eng..

[35] Li Y., Guo Z., Wang L., Zhu Y., Rui S. (2024). Field implementation to resist coastal erosion of sandy slope by eco-friendly methods. Coast. Eng..

[36] Safdar M.U., Mavroulidou M., Gunn M.J., Garelick J., Payne I., Purchase D. (2021). Innovative methods of ground improvement for railway embankment peat fens foundation soil. Géotechnique.

[37] Cheng L., Cord-Ruwisch R. (2012). In situ soil cementation with ureolytic bacteria by surface percolation. Ecol. Eng..

[38] Ghasemi P., Montoya B.M. (2020). Field application of the microbially induced calcium carbonate precipitation on a coastal sandy slope. Geo-Congress 2020.

[39] Tang Y., Lian J., Xu G., Yan Y., Xu H. (2017). Effect of cementation on calcium carbonate precipitation of loose sand resulting from microbial treatment. Trans. Tianjin Univ..

[40] Yang Y., Chu J., Liu H., Cheng L. (2023). Improvement of uniformity of biocemented sand column using CH₃COOH-buffered one-phase-low-pH injection method. Acta Geotech..

[41] Lai H.-J., Cui M.-J., Chu J. (2023). Effect of pH on soil improvement using one-phase-low-pH MICP or EICP biocementation method. Acta Geotech..

[42] Cui M.-J., Lai H.-J., Hoang T., Chu J. (2021). One-phase-low-pH enzyme induced carbonate precipitation (EICP) method for soil improvement. Acta Geotech..

[43] Whiffin V.S., Van Paassen L.A., Harkes M.P. (2007). Microbial carbonate precipitation as a soil improvement technique. Geomicrobiol. J..

[44] Xiao Y., Wang Y., Wang S., Evans T.M., Stuedlein A.W., Chu J., Zhao C., Wu H., Liu H. (2021). Homogeneity and mechanical behaviors of sands improved by a temperature-controlled one-phase MICP method. Acta Geotech..

[45] Do J. (2019). Linguistic Markers of Maternal Focus Within Emotional Conversations: The Role of Depressive Symptoms and Maltreatment. Ph.D. Dissertation.

[46] Karimian A., Hassanlourad M. (2022). Mechanical behaviour of MICP-treated silty sand. Bull. Eng. Geol. Environ..

[47] Najafian Jazi F., Hosseini S.M.M., Ghasemi-Fare O., Rockaway T.D. Volume-change behavior of lime-stabilized expansive soils after experiencing different loading conditions. Proceedings of the IFCEE 2024.

[48] Jazi F.N., Hosseini S.M.M.M., Ghasemi-Fare O., Rockaway T.D. (2024). Experimental evaluation of stress history effect on compressibility characteristics of lime-stabilized expansive soils. Géoméch. Geoengin..

[49] Harkes M.P., Van Paassen L.A., Booster J.L., Whiffin V.S., van Loosdrecht M.C. (2010). Fixation and distribution of bacterial activity in sand to induce carbonate precipitation for ground reinforcement. Ecol. Eng..

[50] Guan D., Zhou Y., Shahin M.A., Tirkolaei H.K., Cheng L. (2023). Assessment of urease enzyme extraction for superior and economic bio-cementation of granular materials using enzyme-induced carbonate precipitation. Acta Geotech..

[51] Rabbani M., Werner J., Fahimi A., Vahidi E. (2025). Innovative pilot-scale process for sustainable rare earth oxide production from coal byproducts: A comprehensive environmental impact assessment. J. Rare Earths.

[52] Zhu T., He R., Hosseini S.M.J., He S., Cheng L., Guo Y., Guo Z. (2024). Influence of precast microbial reinforcement on lateral responses of monopiles. Ocean Eng..

[53] (2016). Standard Test Method for Unconfined Compressive Strength of Cohesive Soil.

[54] Peng J., Liu Z. (2019). Influence of temperature on microbially induced calcium carbonate precipitation for soil treatment. PLoS ONE.

[55] Hosseini S.M.J., Guan D., Cheng L. (2024). Modification of All-in-One Solution for Improvement of 1m Sand Columns. Geomicrobiol. J..

[56] Kooban F., Zadeh S.S., Birgani S.A., Khorshidi M. (2024). Concrete surface crack detection with convolutional-based deep learning models. ESS Open Arch. Eprints.

[57] Zhu Y.-Q., Li Y.-J., Sun X.-Y., Guo Z., Rui S.-J. (2025). A one-phase injection method to improve the strength and uniformity in MICP with polycarboxylic acid added. Acta Geotech..

[58] Liu H., Wei Z., He X., Wang C. (2025). Effect of skim milk powder and injection method on efficiency and uniformity of bio-treated 0.5 m-scale sand column. Sci. China Technol. Sci..

[59] Wang Y., Soga K., DeJong J.T., Kabla A.J. (2019). Microscale visualization of microbial-induced calcium carbonate precipitation processes. J. Geotech. Geoenviron. Eng..

[60] Benini S., Rypniewski W.R., Wilson K.S., Miletti S., Ciurli S., Mangani S. (1999). A new proposal for urease mechanism based on the crystal structures of the native and inhibited enzyme from *Bacillus pasteurii*: Why urea hydrolysis costs two nickels. Structure.

[61] Dadda A., Geindreau C., Emeriault F., Du Roscoat S.R., Garandet A., Sapin L., Filet A.E. (2017). Characterization of microstructural and physical properties changes in biocemented sand using 3D X-ray microtomography. Acta Geotech..

[62] Konopacka-Łyskawa D. (2019). Synthesis Methods and Favorable Conditions for Spherical Vaterite Precipitation: A Review. Curr. Comput. Aided Drug Des..

[63] Meldrum F.C., Hyde S.T. (2001). Morphological Influence of Magnesium and Organic Additives on the Precipitation of Calcite. J. Cryst. Growth.

